# The use of tramadol for cancer-associated pain—a systematic review

**DOI:** 10.1007/s00520-025-10098-4

**Published:** 2025-12-01

**Authors:** Shivani Garlapati, Rachel Alexander, Candice Kaminski, Pavan Challa, Zsuzsanna Nemeth, Eduardo Bruera, Ronald Chow, Elizabeth Prsic

**Affiliations:** 1 Vydehi Institute of Medical Sciences and Research Centre, Bengaluru, Karnataka India; 2 Medstar Georgetown University Hospital, Washington, DC USA; 3 Yale Cancer Center, Yale School of Medicine, Yale University, 333 Cedar Street, P.O. Box 208028, New Haven, CT 06520-8028 USA; 4 University of Texas MD Anderson Cancer Center, Houston, TX USA; 5 Temerty Faculty of Medicine, University of Toronto, Toronto, ON Canada; 6 Centre for Evidence-Based Medicine, University of Oxford, Oxford, UK

**Keywords:** Cancer, Pain, Analgesia, Tramadol

## Abstract

**Background:**

Tramadol has been used for cancer pain and reported in the literature with varying relative effects compared to other analgesics. To our knowledge, there is no comprehensive systematic review documenting the efficacy/effectiveness and safety of tramadol for cancer-associated pain. The aim of this review is to report on the efficacy/effectiveness and safety of tramadol for cancer-associated pain.

**Methods:**

Ovid MEDLINE, Embase, and Cochrane CENTRAL were searched through September 29, 2023. Articles were included if they reported on tramadol in a multi-arm comparative trial, employing either a randomized controlled trial design or an observational study design with a multivariable or propensity-score matched analysis, and reported on efficacy or safety data pertaining to tramadol. A narrative synthesis was conducted to identify common themes across trials of efficacy and safety endpoints.

**Results:**

Eleven studies with 2582 patients were included. Two were cohort studies and nine were randomized controlled trials. There were 20 efficacy endpoints; tramadol was superior in 3, inferior in 4, and neither in 13. There were 80 safety endpoints; tramadol was superior in 9, inferior in 12, and neither in 59.

**Discussion:**

Relative to other analgesics, tramadol is neither superior nor inferior. There may exist a different safety profile and therefore an opportunity to provide individualized patient-centered treatment strategies focused on safety and quality of life.

**Supplementary Information:**

The online version contains supplementary material available at 10.1007/s00520-025-10098-4.

## Introduction

Pain is a prevalent symptom among patients with cancer and frequently diminishes their functional abilities and quality of life (QOL) [1]. In a recent systematic review, including studies from 2014 to 2021, the overall prevalence of pain in patients with cancer was found to be 44.5% [2]. To manage pain, patients are often prescribed multi-modal analgesia as per the WHO analgesic ladder.

Tramadol is a weak opioid and belongs to the second step of the WHO analgesic ladder for the treatment of moderate cancer pain with or without non-opioid analgesics, and with or without adjuvants [3]. Tramadol, being a centrally acting analgesic, has affinity for opioid receptors and influences the action of norepinephrine and serotonin at the synapse. Its dual mechanism of action sets it apart from other step 2 agents [4].

Tramadol has been used for cancer pain and reported in the literature with varying relative effect compared to other analgesics. In a controlled crossover trial by Bono and Cuffari, buprenorphine and tramadol were found to have a similar analgesic effect, although tramadol had a quicker onset of action [5]. In a study by Osipova et al. [6], morphine produced better analgesia than tramadol but was associated with more intensive adverse effects [4, 6].

Tramadol has known adverse effects; a study by Prommer [4] found that the common side effects, in order of decreasing frequency, were dizziness, nausea, constipation, headache, somnolence, vomiting, pruritus, CNS stimulation, sweating, asthenia, dyspepsia, diarrhea, and dry mouth.

Tramadol is metabolized by cytochromes P450 (CYP) 2D6, 2B6, and 3A4, heightening the risk of drug interactions [4]. This risk for drug interactions and its low margin for dose increase due to the risk of severe neurotoxicity have been outlined as major limitations in the 2023 American Society of Clinical Oncology pain guideline [7].

Tramadol has been used for cancer-associated pain with varying levels of efficacy and effectiveness. To our knowledge, there is no comprehensive systematic review documenting the efficacy/effectiveness and safety of tramadol for cancer-associated pain. The aim of this review is to report on the efficacy/effectiveness and safety of tramadol for cancer-associated pain.

## Methods

### Search strategy

With the assistance of a health sciences librarian (ZN), a literature search was conducted in Ovid MEDLINE, Ovid Embase, and Cochrane CENTRAL from database inception through September 29, 2023, inclusively. Searches were adapted for each database and used a combination of keywords and, when applicable, controlled vocabulary. Search concepts were cancer and cancer pain, with restrictions limiting the search to human trials and English-language publications (Appendix).

### Eligibility criteria

After duplication removal and a calibration exercise of ten articles, two review authors (SG, RA, PC) independently screened each article to assess eligibility for this review. Discrepancies were resolved by discussion and consensus or, if needed, the involvement of a third author (RC). Articles were eligible after level 1 title and abstract screening if they reported on human trials of tramadol in the setting of cancer-associated pain in adult (≥ 18 years old) patients. Articles were eligible after level 2 full-text screening if they reported on tramadol in a multi-arm comparative trial, employing either a randomized controlled trial design or an observational study design with a multivariable or propensity-score matched analysis. Finally, articles were included in this review if they had extractable data—either efficacy or safety data, pertaining to tramadol.

### Data extraction

Data extraction was conducted independently and in duplicate by two study authors (SG, RA, CK, PC), and discrepancies were resolved via consensus or involvement of a third author (RC). For each included study, study characteristics (study design, sample size, cancer diagnosis, pain treatment target, comparison arms), patient demographics (mean age, percentage female), relative efficacy, and safety to the comparator arm, as evaluated and reported by studies, were noted. Where clarification was needed for an article, the corresponding authors of the paper were contacted via email for clarification/data and followed up 1 week later if no response was provided. If still no response was provided, the data in question was omitted from this review.

Study quality was assessed using the Cochrane Risk of Bias version 2 for randomized controlled trials [8] and Cochrane Risk of Bias in non-randomized studies of interventions for observational studies [9].

### Synthesis

Narrative synthesis was conducted of common themes across trials. Study characteristics across trials were summarized. Efficacy was narratively reviewed, tabulating the total number of efficacy endpoints across all studies and the conclusions of each endpoint as either favoring tramadol, favoring the control arm, or reporting equivalence. Safety was likewise tabulated by the total number of safety endpoints across all studies and the conclusions of each endpoint as either favoring tramadol, favoring the control arm, or reporting equivalence.

Type I error was set at 0.05. If studies reported the probability value from the statistical testing of their comparison, we reviewed it against our preset type I error to evaluate relative efficacy/safety. If studies did not report the probability value but reported a type I error, we evaluated conservatively for the relative efficacy/safety; if the study’s type I error was set above our type I error (i.e., 0.10) and the study reported superior efficacy/safety for one arm without specifying a probability value, we conservatively evaluated the study as having no difference between tramadol and the comparator arm out of concern that the probability value may be in the range of 0.05 to 0.10 and therefore nonsignificant to our review’s type I error threshold.

## Results

**Fig. 1:**
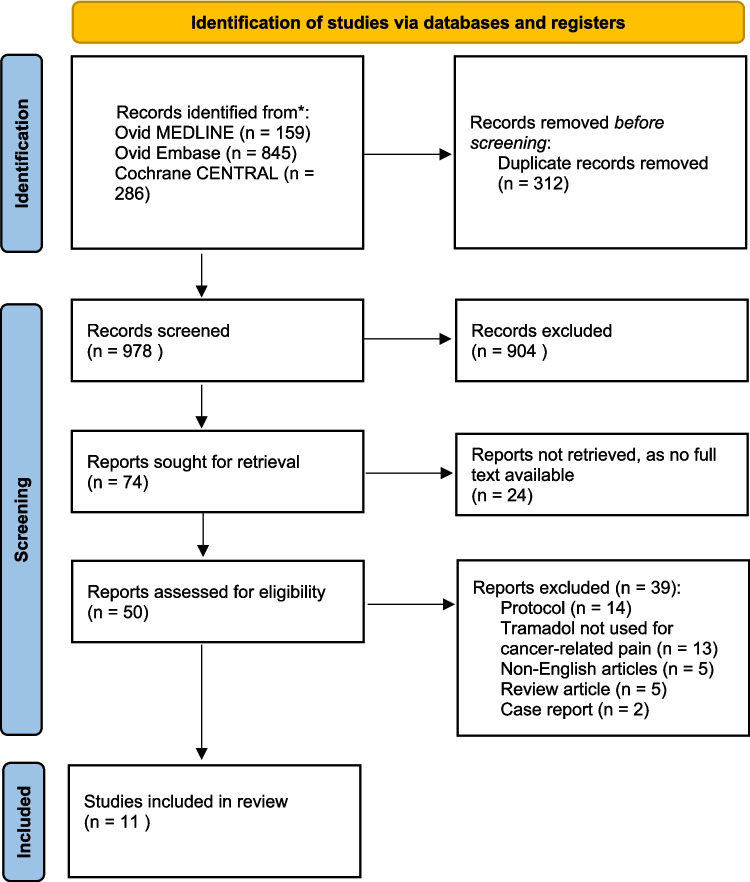
PRISMA diagram

A total of 1290 records were identified through our database searches. After 312 duplicates were removed, 978 records underwent title and abstract screening. Seventy-four full-text studies underwent level 2 full-text screening. Eleven articles were included in this review (Fig. 1).

### Study characteristics

Study demographics are presented in Table 1. Two studies employed a cohort study design, [10, 11] while nine studies employed a randomized control trial design [12–20]. Five studies were open-label design, [11, 13–15, 18] while five used double-blind design [12, 16, 17, 19, 20]. One study which utilized virtual reality in the tramadol group did not specify allocation concealment [10]. The 11 studies compared analgesic effects for a variety of hematologic and solid cancers. Pain targets included somatic, visceral, osseous, mucositis-related, and neuropathic pain. The studies further compared the various adverse effects among the comparison arms. Endpoints were measured in as little as 6 h after administration, up until 4 months after administration.

**Table 1:** Study characteristics

Study	Study design	*n*	Age	% Female	Cancer diagnosis	Comparison arms	Treatment target	Efficacy	Safety
Ahmad et al. 2023 [9]	Cohort Study	80	Mean: 51 ± 10	100	Breast	1.Tramadol + virtual reality 2. Morphine	Moderate to severe cancer pain	Pain: no difference (*p* = 0.85) Anxiety: no difference (*p* = 0.88)	Not reported
Arbaiza et al. 2007 [11]	Randomized controlled trial	36	Mean: 50	61	Breast, lung, prostate, cervical cancer, lymphoma, leukemia	1. Tramadol 2. Placebo	Cancer pain or cancer-related neuropathic pain, tumor-related plexopathy, pain syndrome following surgery, chemotherapy-induced neuropathy, tumor related epidural compression, entrapment of peripheral nerve by tumor mass, pain following herpes zoster	Pain: tramadol superior (*p* < 0.00) Sleep: tramadol superior (*p* < 0.05) Activities of daily living: tramadol superior (*p* < 0.05) Appetite: No difference (*p* > 0.05)	Nausea: tramadol inferior (*p* < 0.05) Vomiting: tramadol inferior (*p* < 0.05) Constipation: tramadol inferior (*p* < 0.05)
Grond et al. 1999 [10]	Cohort study	1,658	Mean: 59 ± 13	46	Head and neck region, gastrointestinal tract, respiratory system, breast, genitourinary system, lymphatic-hematopoietic system, skin, bones, connective tissue	1. Tramadol 2. Morphine	Somatic (bone), somatic (soft tissue), visceral, neuropathic	Pain: no difference (*p* > 0.05)	Constipation: tramadol superior (*p* < 0.05) Neuropsychological symptoms: tramadol superior (*p* < 0.05) Pruritis: tramadol superior (*p* < 0.05) Anorexia: no difference (*p* > 0.05) Dermatologic symptoms: no difference (*p* > 0.05) Diarrhea: no difference (*p* > 0.05) Dyspepsia: no difference (*p* > 0.05) Dyspnea: no difference (*p* > 0.05) Dysphagia: no difference (*p* > 0.05) Insomnia: no difference (*p* > 0.05) Nausea: no difference (*p* > 0.05) Vomiting: no difference (*p* > 0.05) Sweating: no difference (*p* > 0.05) Urinary symptoms: no difference (*p* > 0.05)
Joshi et al. 2021 [12]	Randomized controlled trial	128	Not reported	22	Head and neck cancer	1. Tramadol 2. Diclofenac	Mucositis related	Pain: no difference (*p* = 0.06)	Mucositis: no difference (*p* > 0.05) Dysphagia: no difference (*p* = 1.00) Weight loss: no difference (*p* = 0.72) Nausea: no difference (*p* = 0.47) Vomiting: no difference (*p* = 0.70) Constipation: no difference (*p* = 0.23) Rise in creatinine: no difference (*p* = 0.33) Hyponatremia: no difference (*p* = 0.58) Hypokalemia: no difference (*p* = 0.23) Hypomagnesemia: no difference (*p* = 0.21) SGOT rise: no difference (*p* = 0.63) SGPT rise: no difference (*p* = 0.66) Anemia: no difference (*p* = 0.35) Neutropenia: no difference (*p* = 0.17) Thrombocytopenia: no difference (*p* = 0.83)
Leppert 2001 [13]	Randomized controlled trial	40	Not reported	Not reported	Alimentary system, lung, urinary system, other sites	1. Tramadol 2. Morphine	Visceral, bone, neuropathic, somatic	Visceral pain control: no difference (*p* > 0.05) Bone pain control: no difference (*p* > 0.05) Neuropathic pain control: no difference (*p* > 0.05) Somatic pain control from soft tissues: no difference (*p* > 0.05)	Drowsiness: tramadol superior (*p* < 0.05) Difficulties in passing urine: tramadol superior (*p* < 0.05) Sweating: tramadol superior (*p* < 0.05) Dizziness: tramadol superior (*p* < 0.05)
Leppert 2010 [17]	Randomized controlled trial	30	Mean: 70 ± 9	63	Lung, colon, stomach, palatine tonsil, pharynx, esophagus, gallbladder, pancreas, thyroid and suprarenal glands, kidney, prostate, breast, skin, skin melanoma, myelodysplastic syndrome, Hodgkin disease, ovary, abdominal and pelvic tumors and bone metastases from unknown primary site	1.Tramadol 2. DHC	Nociceptive cancer pain: visceral, somatic, bone	Pain control: tramadol inferior (*p* < 0.01)	Physical functioning: no difference (*p* = 0.24) Role functioning (work): no difference (*p* = 0.20) Cognitive functioning: no difference (*p* = 0.78) Emotional functioning: no difference (*p* = 0.51) Social functioning: no difference (*p* = 0.57) Global quality of life: no difference (*p* = 0.83) Fatigue: no difference (*p* = 0.60) Nausea and vomiting: no difference (*p* = 0.07) Dyspnea: no difference (*p* = 0.35) Sleep: no difference (*p* = 0.06) Appetite: no difference (*p* = 0.17) Constipation: no difference (*p* = 0.64) Diarrhea: no difference (*p* = 0.12) Finances: no difference (*p* = 0.80)
Marinangeli et al., 2007 [14]	Randomized controlled trial	67	Mean: 66 ± 13	42	Respiratory, genitourinary, gastrointestinal/biliary, musculoskeletal	1. Increasing transdermal fentanyl dosage 2. Oral tramadol added to their transdermal fentanyl before each increment of transdermal opioid dosage	Somatic, visceral	Pain control: no difference (*p* > 0.05)	Nausea: no difference (*p* > 0.05) Vomiting: no difference (*p* > 0.05) Constipation: no difference (*p* > 0.05) Diarrhea: no difference (*p* > 0.05) Sedation: no difference (*p* > 0.05) Mental confusion: no difference (*p* > 0.05) Pruritus: no difference (*p* > 0.05)
Rodriguez et al. 2007 [15]	Randomized controlled trial	177	Mean: 60 ± 13	50	Stomach, breast, prostate, lung	1. Tramadol 2. Codeine 3. Hydrocodone	Somatic, visceral, mixed, neuropathic	Pain: no difference (*p* = 0.69)	Vomiting: tramadol inferior (*p* < 0.05) Dizziness: tramadol inferior (*p* < 0.05) Loss of appetite: tramadol inferior (*p* < 0.05) Weakness: tramadol inferior (*p* < 0.05)
Rodriguez et al. 2008 [16]	Randomized controlled trial	118	Mean: 60 ± 14	48	Gastric, breast, prostate, lung	1. Hydrocodone/acetaminophen 2. Tramadol	Somatic, visceral, both somatic and visceral, neuropathic	Analgesic efficacy: no difference (*p* = 0.79)	Nausea: tramadol inferior (*p* = 0.03) Vomiting: tramadol inferior (*p* = 0.02) Dizziness: tramadol inferior (*p* = 0.03) Loss of appetite: tramadol inferior (*p* = 0.02) Weakness: tramadol inferior (*p* = 0.02) Dry mouth: no difference (*p* = 0.61) Constipation: no difference (*p* = 0.62)
Wilder Smith et al. 1994 [18]	Randomized controlled trial	20	Mean: 55	45	Lung, breast, prostate, stomach, non-Hodgkin lymphoma, colon, melanoma	1. Tramadol 2. Morphine	Neurogenic, visceral, osseous	Pain score: no difference (*p* > 0.05)	Nausea: tramadol superior (*p* < 0.05) Constipation: tramadol superior (*p* ≤ 0.05)
Xu et al. 2006 [19]	Randomized controlled trial	230	Mean: 52 ± 21	57	Breast, lung, gastrointestinal, other	1. Tramadol 2. Placebo 3. CKLQ	Somatic, visceral, neuropathic, unknown	Pain: tramadol inferior to CKLQ (*p* < 0.05) Time to pain response: tramadol inferior to CKLQ and placebo (*p* = 0.03) Duration of pain response: tramadol inferior to CKLQ (*p* = 0.00)	Exhaustion: tramadol superior to CKLQ, inferior to placebo (*p* = 0.03) Dizziness: no difference (*p* = 0.54) Nausea: no difference (*p* = 0.33) Vomiting: no difference (*p* = 0.70) Sweating: no difference (*p* = 0.08) Palpitation: no difference (*p* = 0.53) Panting: no difference (*p* = 1.00) Somnolence: no difference (*p* = 1.00) Dysuria: no difference (*p* = 0.50) Constipation: no difference (*p* = 0.09) Diarrhea: no difference (*p* = 0.31)

**Fig. 2:**
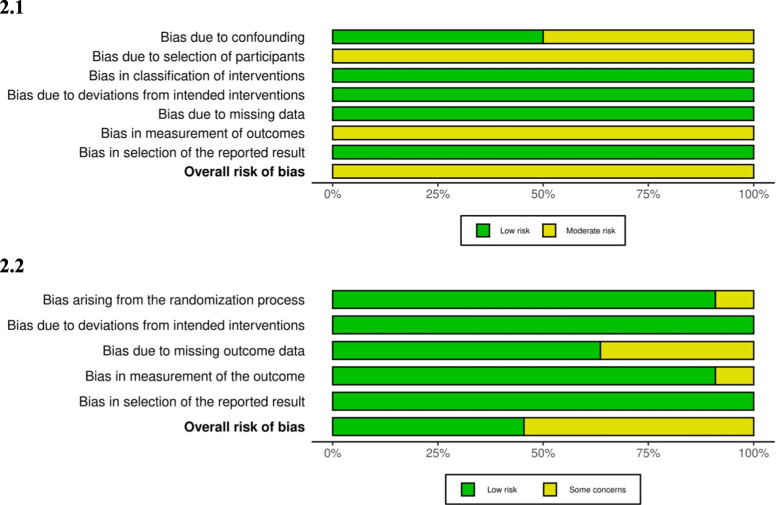
Quality assessment: 2.1 non-randomized trials and 2.2 randomized trials

There was moderate risk/some concern for the overall risk of bias for both cohort studies and over half of the randomized controlled trials (Fig. 2).

### Analgesic efficacy

A total of 20 efficacy endpoints were reported. Tramadol was reported to be superior in 3 efficacy endpoints, inferior in 4 efficacy endpoints, and neither superior nor inferior in 13 efficacy endpoints (Table 1).

Of the 11 studies, all 11 reported on efficacy endpoints. Only one found tramadol to be superior to the comparison arm; tramadol was compared to placebo [12]. Tramadol was also found to be inferior in two studies where it was compared to dihydrocodeine [18] and Compound Keluoqu (CKLQ) [20]. Eight studies reported tramadol to be neither superior nor inferior to comparison arms [10, 11, 13–17, 19]. Four studies compared tramadol to morphine, all of which found tramadol to be neither superior nor inferior for analgesia [10, 11, 14, 19]. Tramadol was also found neither superior nor inferior to codeine and hydrocodone arms, [16] hydrocodone/acetaminophen, [17] and diclofenac [13]. One study incorporated oral tramadol into a pain regimen consisting of escalating doses of transdermal fentanyl; the inclusion of tramadol resulted in a slower rate of escalating doses without a difference in pain control [15].

### Safety endpoints

A total of 80 safety endpoints were reported. Tramadol was reported to be superior to the comparison in 9 safety endpoints, inferior in 12 safety endpoints, and neither superior nor inferior in 59 safety endpoints (Table 1).

Of the 11 studies, all but one [10] reported on safety. Two studies reported tramadol to have superior safety compared to morphine [14, 19]. Two studies reported tramadol to be both superior or no difference to its comparator of morphine [11] and CKLQ [20] in their reporting of multiple safety endpoints. Two studies reported tramadol to have inferior safety compared to placebo [11] and both codeine and hydrocodone [16]. One study reported tramadol to be both inferior or no difference to its comparator of hydrocodone/acetaminophen [17] in their reporting of multiple safety endpoints. Three studies reported no difference between tramadol and diclofenac, [13] DHC [18] and fentanyl [15].

## Discussion

To our knowledge, this is the first systematic review to comprehensively evaluate the relative efficacy/effectiveness of tramadol for the treatment of cancer-related pain. We report that there is conflicting evidence, as summarized across 11 studies with 2582 total patients. The majority of studies report tramadol as neither superior nor inferior to the comparison arm with respect to both efficacy and safety.

It is important to note that the comparison arms vary across studies, with tramadol notably being compared to placebo, morphine, codeine, and hydrocodone for managing cancer-related pain. Only in the trial of tramadol compared to placebo was tramadol superior, which is likely expected as tramadol is a weak opioid and is expected to provide some analgesia compared to no treatment. It is also important to note that the treatment setting for tramadol is very heterogeneous across the literature, with it being used for various types of pain, such as somatic, visceral, neuropathic, and mixed pain syndromes. This heterogeneity and therefore low sample size for each treatment indication may have led to low statistical power and an inability to detect differences between tramadol and its comparator arms. However, this heterogeneity and yet the commonality of tramadol not being superior may also highlight the complexity of managing cancer pain. Cancer pain arises from a complex interplay of inflammatory, neuropathic, and cancer-specific mechanisms [21]. These include neuropathic pain where the cancer cells can infiltrate or compress the sensory nerves; [22] bone remodeling, which can lead to acidosis and sensitize peripheral sensory neurons; [23] peripheral communication between tumor cells, neurons, and surrounding non-neuronal cells, which plays a critical role in initiating and sustaining neuropathic pain related to cancer-directed therapy (e.g., platinums); [21] andthe presence of immune cells recruited to the tumor microenvironment, which can interfere with pain signaling, often exacerbating the perception of pain [24].

Given the lack of difference in efficacy and safety of tramadol to other analgesia, tramadol can be further explored in larger and more rigorous randomized controlled trials. The traditional approach of limiting strong opioids to step III of the WHO analgesic ladder has been increasingly questioned. Stronger opioids are typically reserved for situations where less potent pain medications fail to provide adequate relief, but emerging perspectives challenge this conventional approach. Recent discussions suggest that initiating treatment with a low dose of a higher-potency opioid may be more effective than starting with weaker opioids in specific cases [25]. If tramadol is indeed equivalent in efficacy to higher-potency opioids and yet has an acceptable safety profile, it may be a good alternative to strong opioids. Opioids are associated with constipation [10, 14, 16–18] and neuropsychological symptoms [10]. Tramadol, specifically, may be associated with lower rates of constipation [10, 18] and neuropsychological symptoms [10]. However, the drug is also associated with worse nausea [16], vomiting [15, 16], and dizziness [15, 16] than other opioids.

Furthermore, pain can have a significant impact on quality of life. Tramadol may have varying levels of effects on quality of life, with one study reporting superior outcomes in emotional functioning and cognitive stability, highlighting its potential benefit for patients seeking pain control without compromising cognitive clarity and quality of life. Another study reports poorer general quality of life when compared to other opioids [17]. If, after further study on tramadol, it is determined to be a plausible agent for wider-scale adoption, the different safety profiles of tramadol and other opioids may allow for individualized patient-centered treatment strategies focused on safety and quality of life.

Finally, in pragmatic trials, tramadol has been reported to have several other benefits. Tramadol has been reported to be less addictive than higher-potency opioids [26, 27]. Of final note, when policy change in the USA led to hydrocodone being re-scheduled as a schedule II opioid in 2016, there was a significant increase in the prescription of tramadol [28].

It is important to recognize that a significant proportion of patients with cancer do not receive appropriate analgesia. A systematic review by Greco et al. [29] found that nearly one-third of patients with cancer did not receive analgesic treatment that adequately matched their reported pain levels. This highlights a significant gap in aligning pain management strategies with patient needs, emphasizing the need for understanding the mechanisms underlying cancer pain, improved pain assessment, and individualized care. It is therefore imperative to further explore and optimize analgesic options, including further studying tramadol and other new agents.

There are several limitations of this review. The studies included in this review exhibited substantial heterogeneity in cancer types, pain etiologies, dosing regimens, and outcome measures, which may have led to difficulties with cross-study synthesis and interpretation, impacting the applicability of this review’s findings. We therefore pursued a narrative synthesis approach, maintaining each individual study’s endpoints and instead applying a global type I error of 0.05 to assess individual studies and determine the direction and significance of their results. Also, the majority of the underlying studies have moderate/some concern for bias. Finally, this review was narrative rather than meta-analytical. Ultimately, the results of this review should be interpreted in the context of hypothesis generation, and future studies with more robust protocols and greater statistical power are needed to further investigate the relative efficacy and safety of tramadol.

In conclusion, we report on a systematic review of 11 studies comparing tramadol to other analgesics or placebo for the treatment of cancer pain. We report that the majority of studies report tramadol to be neither superior nor inferior to other analgesics. There may exist a different safety profile and therefore an opportunity to provide individualized patient-centered treatment strategies focused on safety and quality of life. Tramadol may be considered for use in patients with moderate cancer-associated pain, who are at higher risk for opioid dependence and a lower risk of potential adverse events associated with tramadol, including nausea, vomiting, and dizziness. Further large-scale randomized trials are warranted to validate these findings and refine treatment guidelines.

## Supplementary Information

Below is the link to the electronic supplementary material.

ESM 1 Supplementary Material 1 (DOCX 17.3 KB)

## Data Availability

No datasets were generated or analysed during the current study.
