# Supplementary material for: The use of tramadol for cancer-associated pain—a systematic review
Source: Support Care Cancer. 2025 Dec 1;33(12):1152. doi: 10.1007/s00520-025-10098-4 (PMC12669292; doi:10.1007/s00520-025-10098-4)
Supplement: Supplementary file 1 — Supplementary Material 1 (DOCX 17.3 KB) [file 520_2025_10098_MOESM1_ESM.docx]

**Appendix 1.** Search Strategies

Ovid MEDLINE(R) ALL <1946 to September 28, 2023>

1 exp Neoplasms/ 3880206

2 (cancer* or neoplas* or tumor* or tumour* or carcinoma* or adenocarcinoma*).tw,kf. 3895405

3 1 or 2 5045269

4 exp Cancer Pain/ 2363

5 exp Pain Measurement/ 94780

6 exp Pain Management/ 41187

7 exp Pain/ 463020

8 pain.tw,kf. 782617

9 4 or 5 or 6 or 7 or 8 963461

10 3 and 9 140974

11 exp Tramadol/ 3736

12 (tramadol or k315 or ralivia er or rybix or ryzolt or tramahexal or tramake or tramalgin or tramedo or ultram or zamadol or zydol).mp. 6927

13 11 or 12 6927

14 10 and 13 484

15 (randomized controlled trial or controlled clinical trial).pt. or (randomized or randomised or placebo or drug therapy* or randomly or trial or groups).ab. 3740100

16 14 and 15 192

17 exp animals/ not humans.sh. 5158839

18 16 not 17 181

19 limit 18 to english language 159

Embase <1974 to 2023 September 28>

1 exp neoplasm/ 5543014

2 (cancer* or neoplas* or tumor* or tumour* or carcinoma* or adenocarcinoma*).tw,kf. 5154563

3 1 or 2 6660806

4 exp cancer pain/ 23746

5 exp pain measurement/ 30303

6 exp pain/ 1656063

7 pain.tw,kf. 1169457

8 4 or 5 or 6 or 7 1987690

9 3 and 8 427461

10 exp tramadol/ 28981

11 (tramadol or k315 or "ralivia er" or rybix or ryzolt or tramahexal or tramake or tramalgin or tramedo or ultram or zamadol or zydol).mp. 30246

12 10 or 11 30246

13 9 and 12 3637

14 ("randomized controlled trial" or "controlled clinical trial").pt. or (randomized or randomised or placebo or "drug therapy*" or randomly or trial or groups).ab. 5059003

15 13 and 14 918

16 (animal/ or nonhuman/) not human/ 6480443

17 15 not 16 891

18 limit 17 to english language 845

Cochrane

Date Run: 29/09/2023 23:32:03

ID Search Hits

#1 MeSH descriptor: [Neoplasms] explode all trees 112562

#2 (cancer* or neoplas* or tumor* or tumour* or carcinoma* or adenocarcinoma*):ti,ab,kw (Word variations have been searched) 258709

#3 #1 or #2 271221

#4 MeSH descriptor: [Cancer Pain] explode all trees 467

#5 MeSH descriptor: [Pain Measurement] explode all trees 24471

#6 MeSH descriptor: [Pain Management] explode all trees 5108

#7 MeSH descriptor: [Pain] explode all trees 74485

#8 (pain):ti,ab,kw (Word variations have been searched) 235970

#9 #4 or #5 or #6 or #7 or #8 245695

#10 #3 and #9 26687

#11 MeSH descriptor: [Tramadol] explode all trees 1319

#12 (tramadol or k315 or ralivia er or rybix or ryzolt or tramahexal or tramake or tramalgin or tramedo or ultram or zamadol or zydol):ti,ab,kw (Word variations have been searched) 4673

#13 #11 or #12 4673

#14 #10 and #13 351

#15 ((randomized or randomised or placebo or drug therapy* or randomly or trial or groups)):ab (Word variations have been searched) 1439356

#16 #14 AND #15 309

#17 #16 english:la 286
